# Tables for Doctor and Druggist

**Published:** 1891-11

**Authors:** 


Tables for Doctor and Druggist. Comprising: I. Table of Solubilities. II. Table of Reactions and Incompatibles. III. Table of Doses and Uses of Medicines. IV. Table of Specific Gravities. V. Table of Poisons and Antidotes. Compiled by Eli H. Long. M. D., Professor of Materia Medica, Buffalo College of Pharmacy; Adjunct Professor of Materia Medica. Medical Department, University of Buffalo. Pp. 133. George S. Davis, publisher, Detroit. 1891.

We believe that the author has filled a long-felt want. Physicians and pharmacists, for whom this work is primarily intended, have not the time to search through numberless volumes for the information gathered in this little book. By this compilation, the author has placed a ready reference in the hands of the workers, whom, we believe, will thoroughly appreciate the work done for them by Professor Long.

We bespeak for it a bright future, and believe that it will not be long before it will be found on the table of every pharmacist and in the library of every physician.

